# HMGA2 promotes vasculogenic mimicry and tumor aggressiveness by upregulating Twist1 in gastric carcinoma

**DOI:** 10.1038/s41598-017-02494-6

**Published:** 2017-05-22

**Authors:** Junying Sun, Baocun Sun, Ran Sun, Dongwang Zhu, Xiulan Zhao, Yanhui Zhang, Xueyi Dong, Na Che, Jing Li, Fang Liu, Nan Zhao, Yong Wang, Danfang Zhang

**Affiliations:** 1 Department of Pathology, Tianjin Medical University, Tianjin, 300070 China; 2 Department of Pathology, Tianjin General Hospital, Tianjin Medical University, Tianjin, 300052 China; 3 Department of Pathology, Tianjin Cancer Hospital, Tianjin Medical University, Tianjin, 300060 China; 4 Department of Surgery, Tianjin Nankai Hospital, Tianjin, 300100 China; 5 Department of Prosthodontics, Affiliated Stomatological Hospital, Tianjin Medical University, Tianjin, 300070 China

## Abstract

High mobility group protein A2 (HMGA2) is a transcription factor that plays an important role in the invasion and metastasis of gastric carcinoma (GC). The term vasculogenic mimicry (VM) refers to the unique ability of aggressive tumour cells to mimic the pattern of embryonic vasculogenic networks. However, the relationship between HMGA2 and VM formation remains unclear. In the present study, we examined concomitant HMGA2 expression and VM in 228 human GC samples and 4 GC cell lines. Our data indicate that HMGA2 is not only significantly associated with VM formation but also influences the prognosis of patients with gastric carcinoma. Overexpression of HMGA2 significantly increased cell motility, invasiveness, and VM formation both *in vitro* and *in vivo*. A luciferase reporter assay, Co-IP and ChIP demonstrated that HMGA2 induced the expression of Twist1 and VE-cadherin by binding to the Twist1 promoter. Moreover, we observed a decrease in VE-cadherin following Twist1 knockdown in cells overexpressing HMGA2. This study indicates that HMGA2 promotes VM in GC via Twist1-VE-cadherin signalling and influences the prognosis of patients with GC.

## Introduction

Gastric carcinoma (GC) is the second most common cause of cancer-related death in the world, with 42% of those deaths occurring in China^1^. Although treatment ramucirumab, a monoclonal antibody that antagonizes vascular endothelial growth factor receptor(VEGFR)-2, in combination with paclitaxel increases the overall survival of patients with advanced disease^2^, drugs targeting vascular endothelial growth factor (VEGF) signalling such as bevacizumab have failed to improve the survival of patients in phase II and III clinical trials^3, 4^. This finding suggests that VM may represent a mechanism of resistance against angiostatic compounds^5^. In fact, it has been hypothesized that anti-angiogenic therapy might induce the formation of VM^6^.

VM provides a means by which tumours can obtain a blood supply during the early stages of tumourigenesis and represents a pathway by which tumours may become metastatic^7^. It has been demonstrated that patients with tumour-associated VM have a shorter survival time and a higher rate of metastasis than patients without VM^8–10^. While VM has been studied in diverse tumours^8, 10–12^, few studies have focused on its role and clinical significance in GC^9, 13^.

HMGA2 has emerged as a tumour biomarker due to its tumour-specific overexpression in many human cancers. Strong HMGA2 immunopositivity is associated with advanced stage disease and tumour aggressiveness in colorectal^14^, breast^15^, ovarian^16^ and gastric cancers^17^. HMGA2 regulates the expression of several genes by either enhancing or suppressing access to transcription factors, thereby modulating critical cellular processes, such as cell cycle progression, differentiation, and cellular senescence^18–20^. Although the expression and function of HMGA2 have been extensively studied in many cancers, the role of HMGA2 in human GC and its association with VM remain largely uncharacterized. Twist1 is known to promotes VM formation in a variety of tumours^11, 21^ and has been identified as a putative target for HMGA2^22^. Together, these genes are associated with EMT, in which epithelial cells lose many of their epithelial characteristics and present a more mesenchymal phenotype. There are many similarities between VM and EMT, with the former occurring when tumour cells mimic endothelial cells consisting of a type of mesenchymal cell. However, the specific mechanism by which HMGA2 is associated with VM formation and the relationship among HMGA2, VM and Twist1 remains unclear.

In the present study, we examined whether HMGA2 was associated with VM in GC and whether HMGA2 expression and VM impacted the prognosis of GC in a cohort of 228 patients. We similarly investigated the role of HMGA2 in the occurrence of VM in a nude mouse xenograft model. Finally, we determined whether the HMGA2-Twist1-VE-cadherin signalling pathway was critically important for mediating VM in gastric carcinoma.

## Methods

### Patient samples

Two hundred and twenty-eight human GC tissue specimens were collected from patients undergoing surgery at the Tumor Tissue Bank of Tianjin Medical University Cancer Hospital. The histopathological diagnosis was confirmed by trained pathologists. Detailed pathologic and clinical data were collected for all samples. Informed consent was obtained from each patient.

All of the experimental protocols were conducted in accordance with the approved guidelines and were approved by the Ethics Committee of Tianjin Medical University.

### Immunohistochemical and histochemical double-staining methods

Sections were microwaved, blocked, and incubated using a series of antibodies (Table S1). The PicTure PV6000 staining system (Zhongshan Chemical Co.) and Elivision Plus (Zhongshan Chemical Co., Beijing, China) was used. Following IHC staining for CD34/endomucin, the sections were washed with running water for 5 minutes and incubated with periodic acid for 8 min and schiff for 15 min. All sections were counterstained with hematoxylin, dehydrated, and mounted. For the negative controls, Phosphate-buffered saline (PBS) was used in place of the primary antibodies. Sections were evaluated by two independent pathologists who were blinded to the clinical information. Expression of each marker was assessed semi-quantitatively according to the number of cells that stained positive, and the intensity of immunostaining in individual tumour cells^23, 24^. For HMGA2, only immunoreactivity in the nucleus was evaluated. Positivity and negativity for HMGA2 was defined as: negative (−): less than 20% positive nuclear staining from GC cells; positive (+): more than 20% positive nuclear staining from tumour cells.

### Cell lines and cell culture

Cell lines used in this study included: MGC803, MKN45, MKN28, MKN74, and 293T. MKN28 and MKN45 cells were cultured at 37 °C in 5% CO_2_ and saturation humidity in RPMI-1640 medium containing 10% fetal bovine serum (Invitrogen). MKN74, MGC803 and 293T were cultured in Dulbecco’s modified Eagle’s medium supplemented with 10% fetal bovine serum (Invitrogen). MGC803 and MKN45 were obtained from institute of Basic Medical Sciences Chinese Academy of Medical Sciences in 2014. MKN28 was obtained from KeyGEN BioTECH Co. (Nanjing, China) in 2014. MKN74 was obtained from jenniobio Biotechnology Co. (Guangzhou, China) in 2015. 293T was obtained from Zhongshan Hospital Affiliated to Fudan University (Shanghai, China) in 2014.

### Lentiviral constructs and cell infection

Full-length HMGA2 complementary DNA (cDNA), HMGA2 gene expression inhibitor plasmid and the respective empty vector plasmids were purchased from Gene Copoeia (US). The pEZ-Lv201 vector was used for gene transfer of MKN74 and MKN28 cells to overexpress HMGA2, and the psi-LVRU6GP vector was used for HMGA2 silencing in MGC803 and MKN45 cells.

The small interfering RNA (si RNA) kit (pGP-Twist1-shRNA) was also purchased from Gene Copoeia (US). The target sequence (Twist1-shRNA1: GGTAACAATCAGAGGAACTAT and Twist1-shRNA2: GCAAGATTCAGACCCTCAAGC) were used to downregulate Twist1 *in vitro*.

Lentiviruses were produced via transient transfection of 293T cells, and the virus suspension was used to infect target cells.

### Semi-quantitative RT-PCR

Total cellular RNA was extracted using the TRIzol reagent according to the manufacturer’s instructions. For semi-quantitative RT-PCR, 2 ug of total RNA was reverse transcribed into cDNA in a 25 uL reaction using the QuantScript RT Kit (Tiangen Biotech). Semi-quantitative RT-PCR was performed according to the recommended thermal profile: 95 °C for 5 minutes(preincubation), followed by 30 cycles at 95 °C for 30 seconds (denaturation), 60 °C for 1 minute (annealing), and 72 °C for 30 seconds (elongation). The amplified products were subjected to electrophoresis on a 1% agarose gel containing ethidium bromide (Bio-Rad). The expression of GAPDH was used to examine the integrity of the RNA in each sample and to standardize the amount of cDNA added to each PCR tube. The primers used for semi-quantitative RT-PCR are described in Table S2.

### Immunofluorescent staining

Cells were cultured on coverslips 1 day prior to staining. Cells were fixed with cold methanol for 10 minutes and blocked with 1% BSA. The coverslips were incubated overnight, at 4 °C with primary antibodies (Table S1) and incubated with secondary antibodies at 37 °C for 1 hour. FITC- and TRITC-conjugated mouse and rabbit Ig G antibodies (Santa Cruz) were used to label the cells for immunofluorescence assays. Following immunolabeling, the cells were washed, stained with DAPI (Sigma), mounted, and viewed under a fluorescence microscope (Nikon, Japan).

### Western blot

Cells were lysed and then transferred to polyvinylidene difluoride membranes (Millipore). The membranes were blocked and incubated with primary antibodies (Table S1) at 4 °C overnight, followed by incubation with a secondary antibody (1:2000, Cat.#sc-2055, Cat.#sc-2004, Santa Cruz, CA, USA). The membranes were developed using an enhanced chemiluminescence (ECL) detection kit (Amersham Pharmacia Biotech, Piscataway, NJ, USA). Equal sample loading was confirmed by probing the membranes with GAPDH. The intensity of the protein bands was determined via densitometry using ImageJ system.

### Gelatin zymography

All media were collected and subjected to SDS-PAGE using 10% polyacrylamide gels containing 0.01% w/v gelatin. Following electrophoresis, gels were equilibrated in 2.5% Triton X-100 and incubated in 50 mmol/L Tris-HCl (pH 7.5), 10 mmol/L CaCl_2_, 150 mmol/L NaCl and 1 mmol/L ZnCl_2_, for 42 hours at 37 °C. These were stained with Coomassie Brilliant Blue G250 and destained until the wash buffer became clear with apparent cleared zones associated with MMP activity.

### Co-Immunoprecipitation

The Co-IP products of HMGA2/Twist1 were immunoprecipitated from cell lysates using the Pierce Crosslink Immunoprecipitation Kit (Thermo Fisher Scientific, 26147) with HMGA2 (#5269, Cell Signaling, USA), Twist1, (SC-15393 X, Santa Cruz, USA). IgG (AB171870, Abcam, UK) was used as control. Input and immunoprecipitated proteins were analyzed by western blot.

### Luciferase reporter assay

The Twist1 promoter was PCR-amplified from human genomic DNA and cloned into the pEZX-LvPG04 Luciferase Reporter Vector (Genecopoeia). 293T cells were transfected with Twist1 promoter plasmids or the control plasmid and HMGA2 plasmid. 48 hours following transfection, luciferase activity was analyzed using the Secrete-Pair^TM^ Dual Luminescence Assay Kits (Gene Copoeia^TM^). The results were obtained from three independent experiments performed in duplicate.

### Chromatin immunoprecipitation (ChIP)-qPCR

Chromatin immunoprecipitation (ChIP) assay was performed as previously described^25^. Real-time PCR was conducted by SYBR Green-based detection method (Applied Biosystems) using equal amounts of ChIP and diluted input DNAs. Primer sequences are listed in the Table S3. Antibodies used for the ChIP assays included anti-HMGA2 (GTX100519, GeneTex, USA) and irrelevant IgG control antibody (AB171870, Abcam, UK).

### Cell invasion and migration assay

Cell invasion and migration assay was performed using transwell cell culture inserts (Invitrogen, Carlsbad, CA, USA). For invasion assays, transwell chambers were first coated with matrigel (1 mg/ml; BD Biosciences). Cells (1 × 10^5^) were seeded into the upper chambers in serum-free media, while the lower chambers contained media with 10% FBS as a chemo attractant. The cells were incubated for 48 hours at 37 °C, 5% CO_2_. Cells that invaded the Matrigel matrix were fixed with methanol and stained with 0.5% crystal violet. The number of invading cells was counted using an inverted light microscope (Nikon). For the migration assay, the cells were used as transwell cell inserts without Matrigel matrix, and a transwell assay was performed according to the above protocol. The numbers of invading cells at 24 hours were counted. Each experiment was performed in triplicate.

### Three-dimensional (3D) cultures

A 96-well culture plate was coated with 20 μl/well of Matrigel matrix (BD Biosciences), which was allowed to polymerize for 1 hour at 37 °C. Then a cell suspension (1 × 10^5^ cells/well) in regular medium was seeded on top of the gel, and three wells were provided for each group. The cells were continuously observed for 12–48 hours in the 3D culture system. Cells were photographed using a phase contrast microscope.

### Animal experiments

For the subcutaneous xenograft model, MKN74 cells (1 × 10^7^) and MGC803 cells (1 × 10^7^) (stably transfected with HMGA2 expression plasmid, the HMGA2 sh-RNA plasmid, and the corresponding control vector) were suspended in 100 μl of PBS and then subcutaneously injected into the upper right flank region of female BALB/c-nu/nu mice at 3–4 weeks of age. Seven days following tumor cell engraftment, tumour growth was evaluated by measuring the length and width of the tumour mass at the inoculation site. Tumour volume was monitored weekly using digital calipers and calculated using the following formula: TV = 1/2 × a × b^2^ (where a is the length and b is the width of tumour). After 4–5 weeks, the mice were scarified, and tumours were dissected, fixed in formalin, and embedded in paraffin. All of the experimental protocols were conducted in accordance with our Institutional Animal Care and Use Committee (IACUC) guidelines and were approved by the Tianjin Medical University IACUC committee.

### Statistical analysis

The data were analyzed with SPSS 22.0 (SPSS Inc., Chicago, IL, USA). *P* < 0.05 was considered statistically significant. All statistical analyses were performed using ANOVA or a two-tailed Student’s *t-*test to compare the data. Survival curves were calculated using the Kaplan-Meier method.

## Results

### HMGA2 is associated with VM in GC patients and predicts an adverse clinical outcome

**Figure 1:**
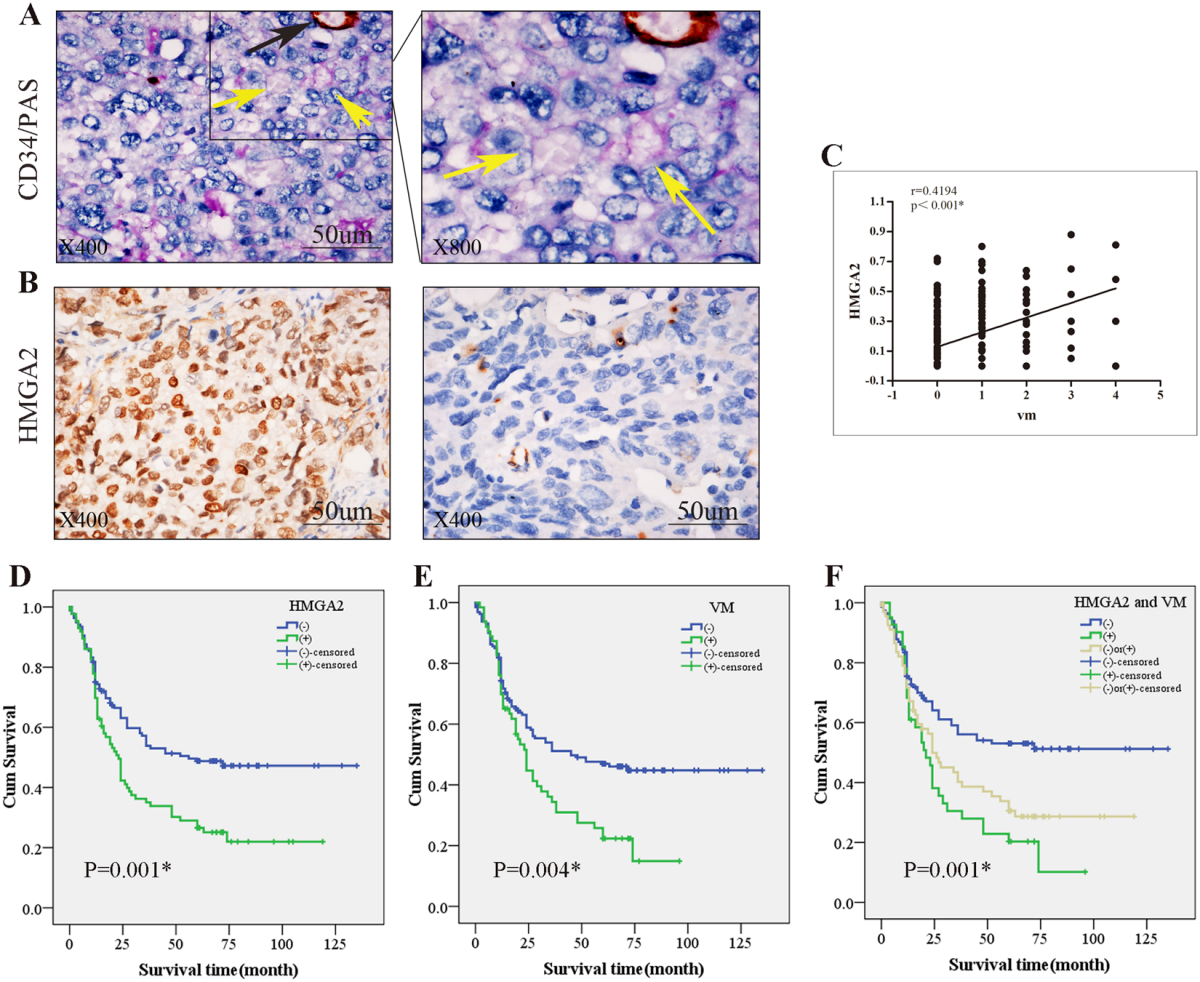
The correlation of HMGA2 expression with VM in clinical samples. (**A**) Phenomena of VM (yellow arrow, CD34-negative and PAS positive) and EDV (black arrow, CD34-positive and PAS-positive) in gastric carcinoma specimens; (**B**) IHC staining of HMGA2, the left for positive and the right for negative nuclear expression (×400, bar 50 um); (**C**) Quantum analysis of the correlation of HMGA2 with VM. A positive correlation between HMGA2 and VM using the Pearson correlation was observed (*P < 0.001, r = 0.4194) (**D**,**E**,**F**) Kaplan-Meier survival curve demonstrated that HMGA2 expression (**D**), VM (**E**), and a combination of HMGA2 expression and VM density (**F**) are significantly related to poor prognosis. (Log Rank χ^2^ = 10.693, *P* = 0.001; Log Rank χ^2^ = 8.413, *P* = 0.004; Log Rank χ^2^ = 14.494, *P* = 0.001).

To identify the correlation between HMGA2 and VM in GC patients, we evaluated HMGA2 expression and VM in a cohort of 228 gastric carcinoma specimens collected from the Cancer Hospital of Tianjin Medical University. Of these, 88(38.6%) samples were positive for HMGA2 (Table 1 and Fig. 1B).

**Table 1:** The correlation of HMGA2 and VM with the clinicopathological parameter of gastric carcinoma.

Variant		Tissue samples		χ^2^	*P* Value	Tissue samples		χ^2^	*P* Value
		HMGA2	Non-HMGA2			VM	Non-VM		
Age(years)	<60	34	71	3.173	0.078	24	81	3.05	0.105
	≥60	54	69			41	82		
Sex	Male	22	52	3.634	0.061	47	107	0.941	0.352
	Female	66	88			18	56		
Tumor size(cm)	<3	31	65	2.781	0.101	26	70	0.165	0.767
	≥3	57	75			39	93		
Histological differentiation	I/II	32	85	12.825	**	26	91	4.66	0.040*
	III/IV	56	55			39	72		
Stage	I/II	26	73	11.231	0.001*	15	84	15.317	**
	III/IV	62	67			50	79		
Metastasis and recurrence	Present	63	42	37.623	**	44	61	17.137	**
	Absent	25	98			21	102		

*P < 0.05, **P < 0.001.

Tube cavities lined with PAS-positive, CD34-negative tumour cells and red blood cells observed within cavities were deemed VM channels^26, 27^ (yellow arrows, Fig. 1A). Channels positive for both PAS and CD34 were defined as EDVs (black arrows, Fig. 1A). Notably, our results demonstrated that high HMGA2 overexpression and VM were both correlated with the tumour differentiation, stage, metastasis and poor prognosis (*P* < 0.05, Table 1, and Fig. 1D,E).

In VM-positive cases, HMGA2 was identified in 42 (64.6%) patients, and we also observed a significant correlation of HMGA2 expression with the formation of VM (*P* < 0.001, r = 0.4194 Fig. 1C). To assess whether HMGA2 and VM are predictors of survival, we performed Kaplan-Meier analyses. The results revealed that the overall survival rate was significantly lower in patients whose tumours showed double positive staining for HMGA2 and VM than those with negative expression for either of these factors (Fig. 1F).

### HMGA2 promotes VM formation, migration and invasion of GC cells *in vitro*

**Figure 2:**
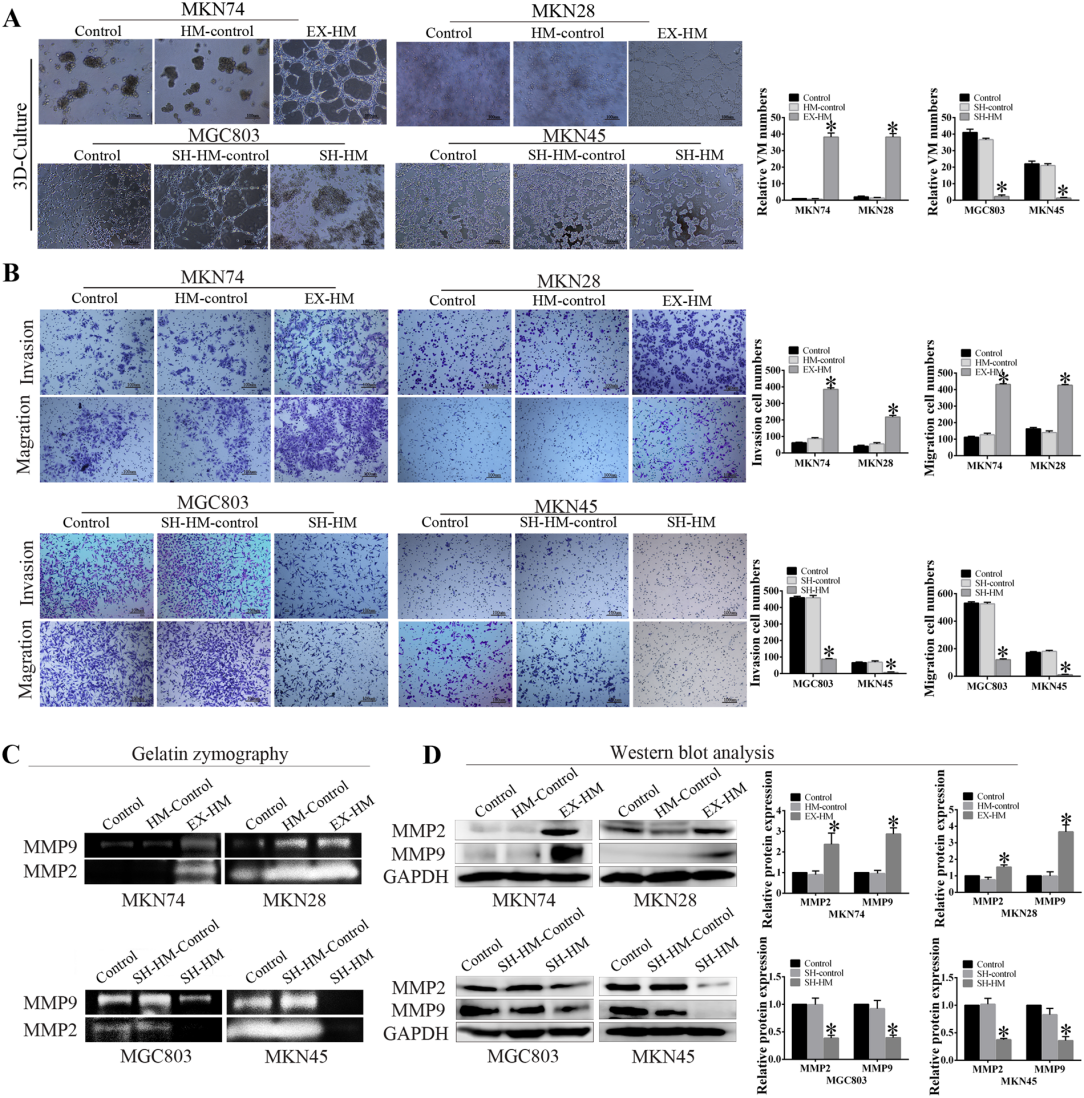
HMGA2 promotes VM formation, invasion and migration of GC cells *in vitro*. (**A**) HMGA2 overexpression facilitated the development of VM tubes whereas HMGA2 silencing inhibited VM formation on Matrigel (bar 100 um, *P < 0.05 n = 3); (**B**) The invasion and migration abilities of gastric carcinoma cells were increased following HMGA2 overexpression, and decreased by HMGA2 silencing (bar 100 um *P < 0.001); (**C**) Gelatin zymography analysis the activity of MMP. (**D**) Western blot showed the protein expression of MMP2 and MMP9 in GC cells. *P < 0.001.

To investigate whether HMGA2 contributes to VM, we compared the protein expression of HMGA2 in various GC cell lines (Fig. S1A) and generated cell lines that overexpressed or inhibited HMGA2. We performed 3D-culturing to examine the function of HMGA2 on VM *in vitro*. Our results demonstrated that HMGA2 overexpression facilitated the development of VM tubes in MKN74/MKN28 cells, whereas HMGA2 silencing in MGC803/MKN45 cells inhibited VM formation on Matrigel (*P* < 0.05, Fig. 2A). To further demonstrate that the tumour cells that formed the VM were overexpressing HMGA2, we performed immunofluorescent staining in 3D-cultured cells. The results showed that cells formed tubular structured that expressed HMGA2 (Fig. S1B).

VM formation has been associated with cell migration and invasion, thus, to further explore HMGA2-associated VM in GC, we performed invasion and migration assays. Our results demonstrated that HMGA2 knockdown significantly decreased the abilities of MGC803 and MKN45 cells to invade and migrate, whereas HMGA2 overexpression had the inverse effect on MKN74 and MKN28 cells. (Fig. 2B, *P* < 0.05 for all).

Not only did we observe a change in cell function but we also detected changes at the molecular level. Gelatin zymography analysis revealed that MMP activity was increased following HMGA2 overexpression (Fig. 2C). Meanwhile, the results of our Western blot studies demonstrate that HMGA2 overexpression led to increased expression of MMP2 and MMP9 (Fig. 2D). Simultaneously, in MGC803 and MKN45 with HMGA2 knockdown, MMP2 and MMP9 expression was reduced. MMP2 and MMP9 activity was not only involved in invasion and metastasis but also associated with VM formation^11, 28^. Taken together, our data indicate that HMGA2 increased VM formation as well as the migratory and invasive abilities in human GC cell lines.

### HMGA2 upregulates Twist1 and VE-cadherin expression in GC cell lines

**Figure 3:**
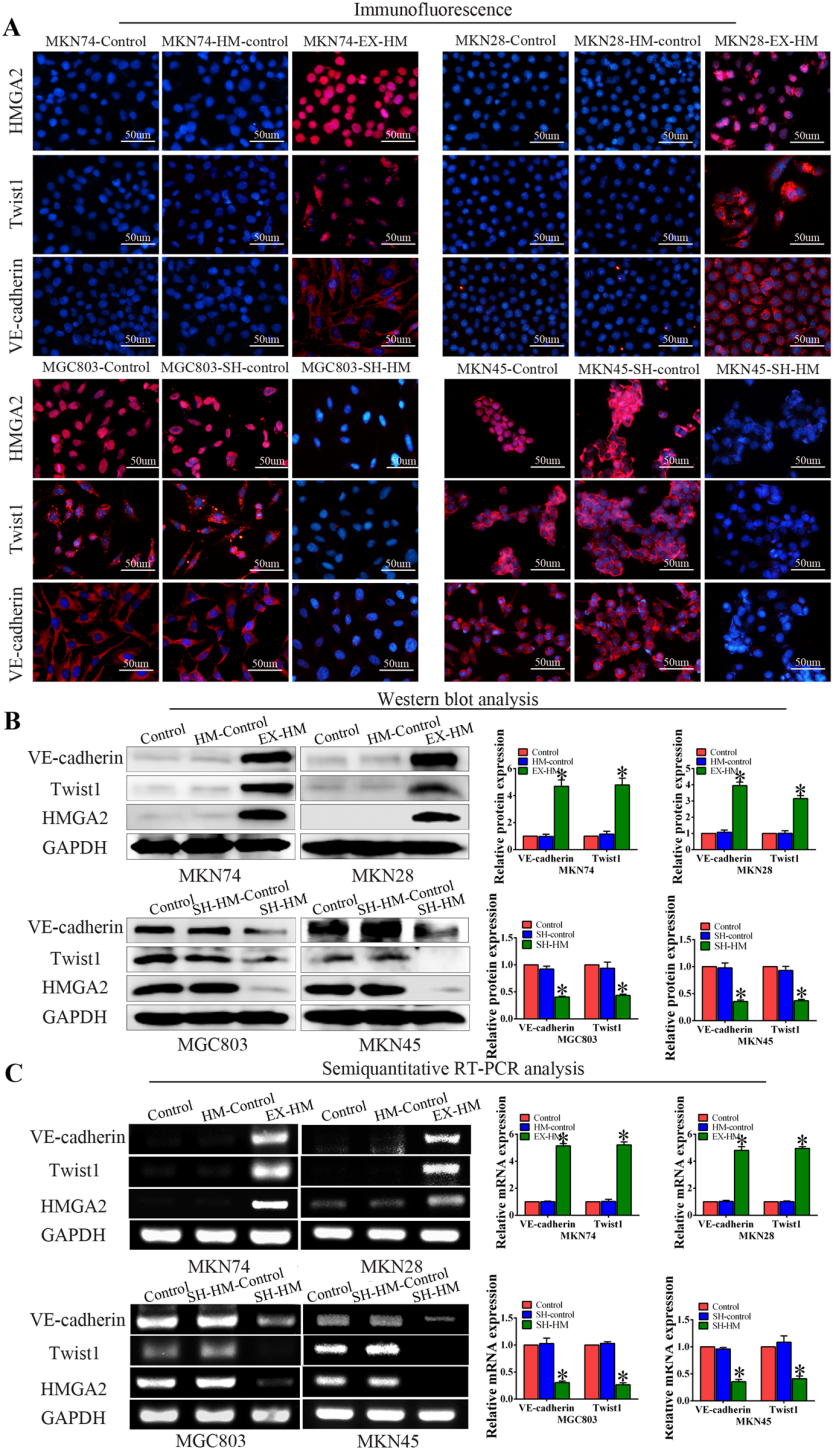
HMGA2 upregulates Twist1 and VE-cadherin expression in GC cell lines. (**A**) Immunofluorescence staining. Overexpression of HMGA2 increased the expression of Twist1 and VE-cadherin, while HMGA2 silencing inhibited Twist1 and VE-cadherin expression (bar, 50 um). Western blot (**B**) and semi-quantitative RT-PCR (**C**) were performed to analyze the expression of Twist1 and VE-cadherin in GC cells (*P < 0.05). The results show that Overexpression HMGA2 induced upregulation of Twist1 and VE-cadherin expression, the inverse change were obtained with the knockdown of HMGA2.

Previous reports have indicated that expression of Twist1, which is regulated by HMGA2^29^, is biologically and clinically associated with VM in tumours^21, 30^. To investigate whether Twist1 was involved in HMGA2-induced VM in GC, we examined the expression levels of Twist1 using Western blotting, semi-quantitative RT-PCR and immunofluorescence. The Western blot (Fig. 3B) and RT-PCR (Fig. 3C) results confirmed that increased HMGA2 expression upregulated Twist1 expression. The gray analysis showed that the differences were statistically significant. Furthermore, the immunofluorescence results revealed that reduced expression of Twist1 in MGC803 and MKN45 cells occurs following HMGA2 knockdown. Conversely, MKN28 and MKN74 cells overexpressing HMGA2 demonstrated upregulated levels of Twist1 expression (Fig. 3A).

Next, we detected the expression of VE-cadherin to analyse the correlation between HMGA2 and VE-cadherin not only because Twist1 promotes VM formation by targeting its promoter but also due to its frequent overexpression in tumour tissues and its importance in both tumour invasion and VM formation^31^. The results of the Western blot and semi-quantitative RT-PCR showed that VE-cadherin expression was upregulated following overexpression of HMGA2 and downregulated following shRNA-mediated suppression of HMGA2 in GC cell lines (Fig. 3B,C). The immunofluorescence results demonstrated that VE-cadherin expression was higher in the HMGA2-overexpressing group than the control group, whereas its expression was lower in HMGA2-downregulated group (Fig. 3A). These findings suggest that HMGA2 induces VM by upregulating Twist1 and VE-cadherin in GC cell lines.

### HMGA2 directly targets Twist1 to promote VM in GC

**Figure 4:**
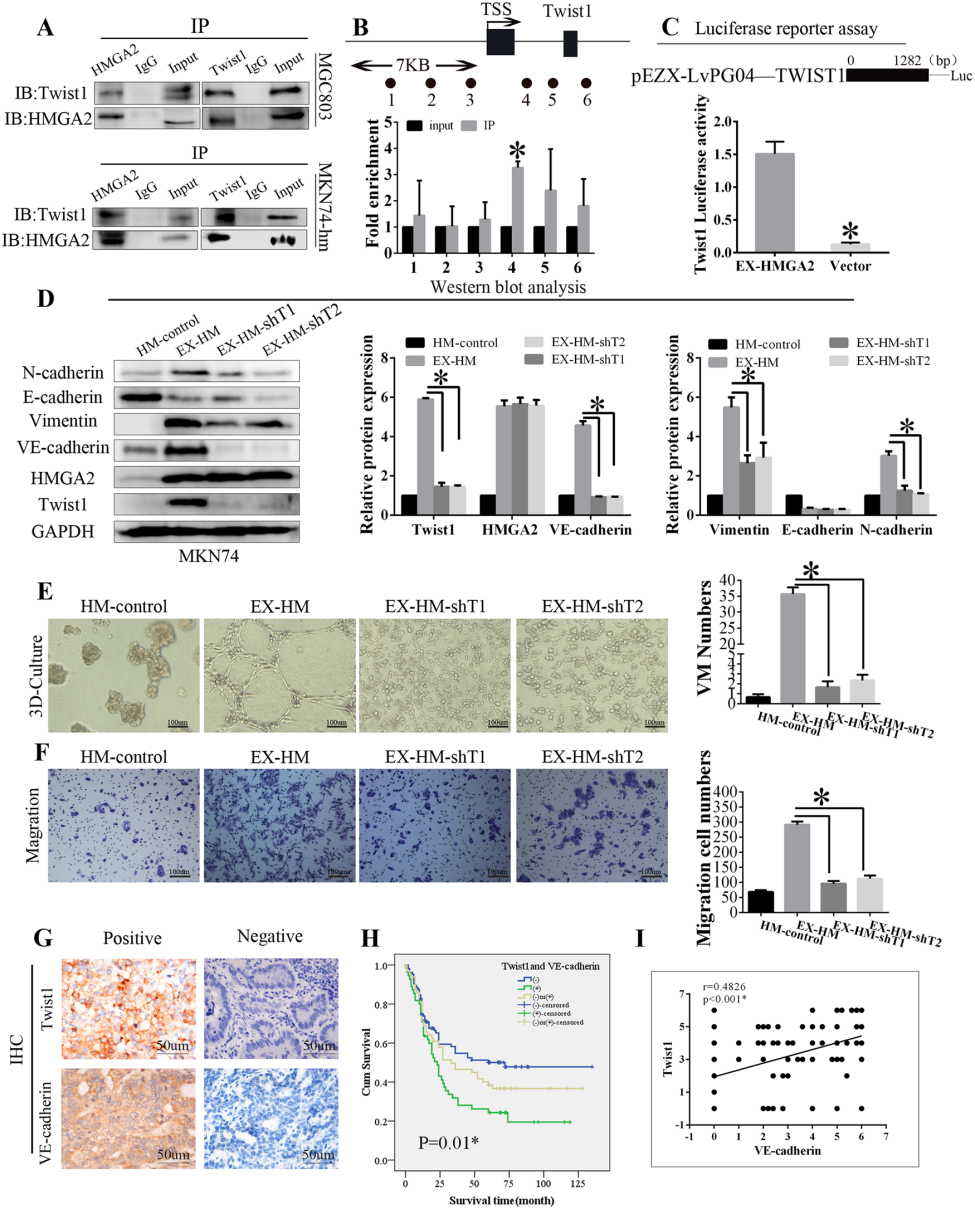
HMGA2 directly targets Twist1 to promote VM in GC. (**A**) HMGA2 interacted with Twist1 in MGC803 and MKN74 cell line with HMGA2 overexpression. (**B**) ChIP was performed on MKN74 cells transfected with pc DNA-HMGA2. The precipitated chromatin was PCR-amplified with the use of specific primers in the Twist1 promoter (Supplementary Table S3) as indicated by black dots. Bar graphs show fold enrichment of HMGA2 binding of Twist1 promoter. Relative enrichment compared to irrelative antibody control is shown. The mean ± SD of three determinations is shown. (**C**) Luciferase activity assays in 293T cells showed the enhancement of Twist1 promoter activity by HMGA2 (*P < 0.05). (**D**) Western blot showed the protein expression of HMGA2, Twist1, VE-cadherin, vimentin, E-cadherin, N-cadherin when Twist1 knockdown in MKN74 cells with HMGA2 overexpression (*P < 0.05). 3D culture (**E**) and migration (**F**) showed Twist1-konckdown decreased VM formation and migration in MKN74 cells with HMGA2 overexpression. Scale bar represents 100 um, *P < 0.05. (**G**) IHC staining of Twist1 and VE-cadherin in GC samples (×200, bar 50 um); (**H**) Kaplan-Meier survival analysis of GC patients categorized by a combination of Twist1 and VE-cadherin (*P < 0.05). (**I**) A positive correlation between Twist1 and VE-cadherin using the Pearson correlation (r = 0.4826 *P < 0.001).

To investigate how HMGA2 affected the expression of Twist1, a luciferase reporter assay was performed. The results demonstrated that overexpression of HMGA2 increased the luciferase activity driven by the Twist1 promoter (*P* = 0.024, Fig. 4C). Meanwhile, the Co-IP analysis indicated that HMGA2 could directly bind Twist1 in MGC803 cells with endogenous overexpression of HMGA2 as well as in MKN74-hmga2 cells with exogenous expression of HMGA2 (Fig. 4A).

To further clarify the regulatory mechanism of Twist1 by HMGA2, we conducted chromatin immunoprecipitation (ChIP)-qPCR. The primers used in this experiment targeted HMGA2 binding elements in the promoter regions of Twist1 (Primer sequences are listed in Table S3). Six primers pairs were designed. q-RT-PCR analysis revealed that only the primer 4 showed high amplification efficiency (Fig. 4B), which suggested that HMGA2 could directly bind to the promoter region of Twist1 and regulate its expression.

To further demonstrate that HMGA2 promotes VM formation by targeting Twist1, we silenced Twist1 expression in the MKN74-hmga2 cell line (MKN74-hm-sht). The 3D-culture was performed to detect VM formation in MKN74-hm-sht cells. The results showed that silencing Twist1 significantly reduced tube formation (Fig. 4E). Concomitant with this observation was a significant decrease in cell migration (Fig. 4F). Moreover, we also explored the expression of N-cadherin, E-cadherin, vimentin, and VE-cadherin in MKN74-hm-sht cells (Fig. 4D). Our results demonstrate that silencing Twist1 reduced the protein expression of Twist1,VE-cadherin, N-cadherin, vimentin compared with the control group, but the HMGA2 and E-cadherin expression did not increased.

We further analysed Twist1 expression in human GC tissues as well as its association with VE-cadherin. The results of the IHC staining showed that Twist1 expression was correlated with VE-cadherin (Fig. 4G,I). The Kaplan-Meier survival analysis indicated that patients with elevated Twist1 and VE-cadherin expression have a poor prognosis (Fig. 4H). Meanwhile, we analysed the correlation among HMGA2, Twist1 and VE-cadherin expression in human GC, and the results demonstrated that their expression was linearly correlated (Fig. S1C).

### HMGA2 is associated with the EMT signalling

EMT has been proposed as a key process in cancer progression, in which epithelial cells acquire mesenchymal properties and exhibit decreased cell-matrix adhesion. Notably, there are many common pathways regulating EMT and VM. To better understand the mechanism by which HMGA2 promotes VM formation, the expression levels of EMT-associated factors were evaluated.

**Figure 5:**
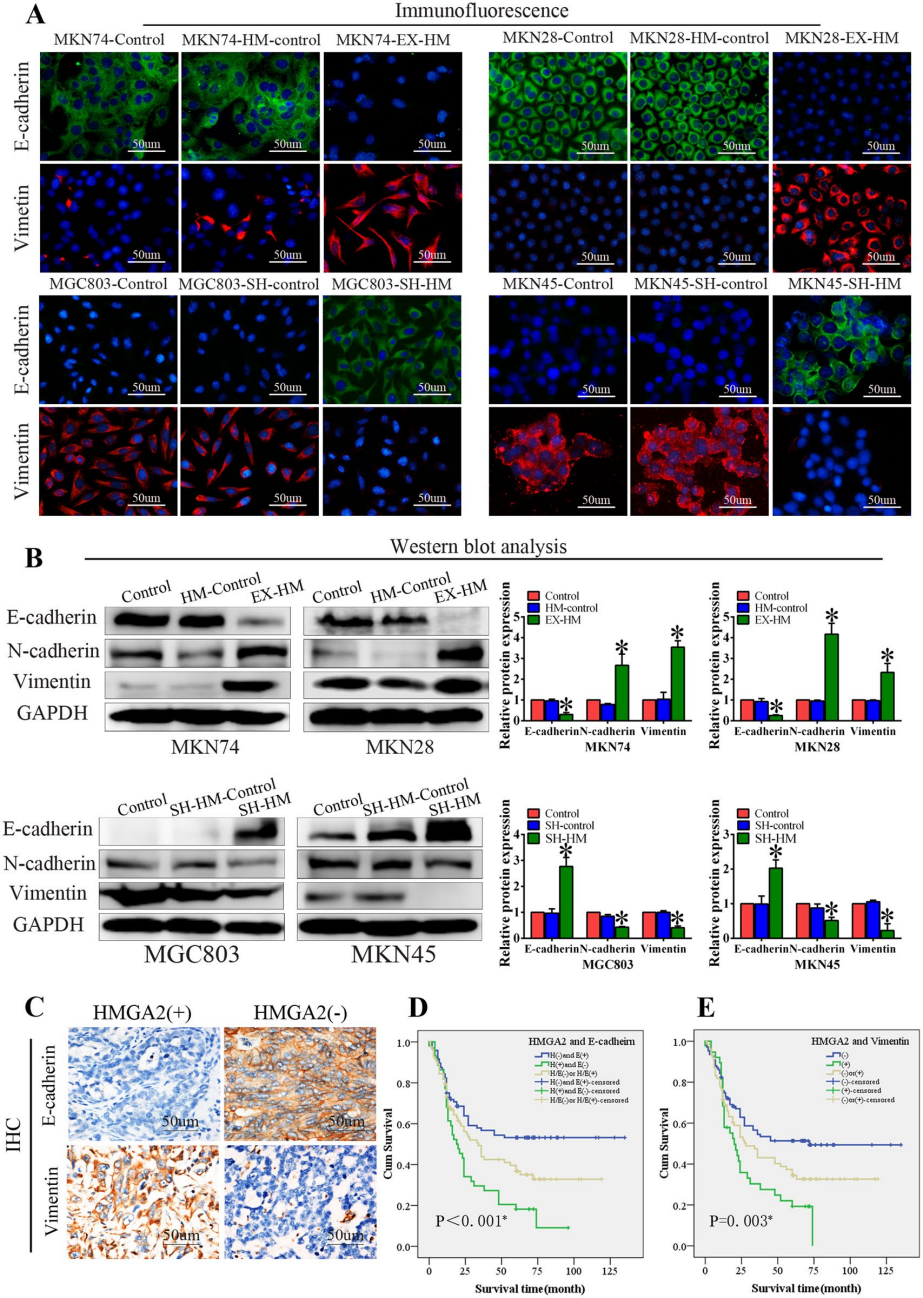
HMGA2 is associated with the EMT signaling. MKN74 and MKN28 cells stably transfected with HMGA2 expression plasmid and MGC803 and MKN45 cells stably transfected with HMGA2 sh-RNA plasmid. (**A**) Immunofluorescence staining. Overexpression of HMGA2 increased the protein expression of vimentin and inactivated the expression of E-cadherin and HMGA2 silencing inhibited vimentin expression and upregulated E-cadherin expression (bar, 50 um). (**B**) Western blot showed that expression of E-cadherin and vimentin, N-cadherin in GC cell lines (*P < 0.05). (**C**) IHC staining showed the expression of E-cadherin and vimentin in GC samples (×200, bar 50 um). (**D**,**E**) Kaplan-Meier survival analysis of GC patients categorized by a combination of HMGA2 and E-cadherin (**D**), vimentin (**E**) (*P < 0.05).

The immunofluorescence studies demonstrated that HMGA2 significantly enhanced the expression of the mesenchymal marker vimentin and decreased the expression of the epithelial marker E-cadherin in MKN74 and MKN28 cells that overexpressed HMGA2 (Fig. 5A). These changes in E-cadherin and vimentin protein expression were verified by Western blot (Fig. 5B). The protein expression of N-cadherin, another mesenchymal marker, was also increased by HMGA2. Conversely, E-cadherin expression was enhanced but the vimentin and N-cadherin expression levels were suppressed in MGC803 and MKN45 cells transfected with the sh-HMGA2 plasmid.

We also evaluated the expression of E-cadherin and vimentin across our cohort of GC specimens and confirmed that E-cadherin expression was lower and vimentin expression was higher in HMGA2-positive GC patients than those who were HMGA2-negative (Fig. 5C). A survival analysis of HMGA2 and E-cadherin expression demonstrated that patients with tumours with high levels of HMGA2 and low levels of E-cadherin have a shorter survival time compared with patients with low HMGA2 expressing and high E-cadherin expressing tumours (Fig. 5D); meanwhile, GC patients with high levels of HMGA2 and vimentin expression have a poor prognosis (Fig. 5E).

### HMGA2 promotes tumour growth and vasculogenic mimicry in a xenograft tumour model

To validate the function of HMGA2 *in vivo*, MKN74 cells stably transfected with an HMGA2-expression plasmid and MGC803 cells stably transfected with an HMGA2 inhibitor were subcutaneously injected into BALB/c-nu/nu mice.

**Figure 6:**
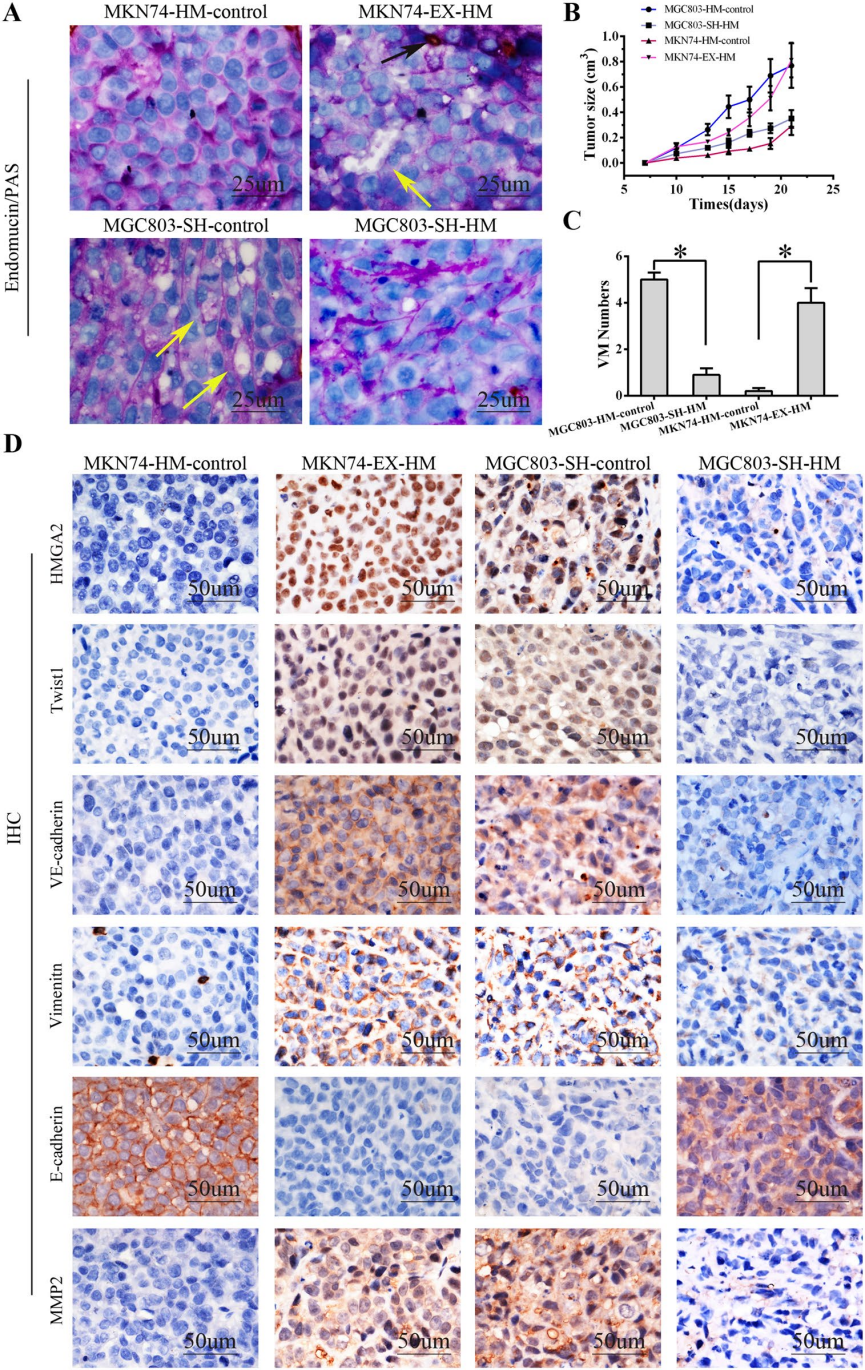
HMGA2 promotes tumour growth and vasculogenic mimicry in a xenograft tumour model. MKN74 cells stably transfected with HMGA2 expression plasmid and MGC803 cells stably transfected with the HMGA2 sh-RNA plasmid were subcutaneously injected into BALB/c-nu/nu mice. (**A**) EDV with Endomucin expression (the black arrow); VM channels with PAS-positive substance and negative Endomucin expression, and red cells remained visible at the center of the VM channels (the yellow arrow) (bar 25 um); (**B**) Tumour growth curve showed HMGA2 overexpression promotes tumor growth; (**C**) Tumours with HMGA2 overexpression have more VM tubes; (**D**) IHC staining showed that tumours with higher HMGA2 expression had elevated Twist1, VE-cadherin, vimentin, MMP2 expression and loss of E-cadherin proteins (×400, bar 50 um).

The effects of HMGA2 on tumour growth in nude mice are reflected by growth curves. The growth curves showed that murine xenografted with cells with high HMGA2 expression developed faster than those with low HMGA2 expression (*P* < 0.05, Fig. 6B).

To evaluate the relationship between HMGA2 expression and VM *in vivo*, we performed endomucin/PAS double staining. VM (Fig. 6A, yellow arrow) formation increased in xenografted MKN74 tumors overexpressing HMGA2 and decreased in xenografted MGC803 tumors with silenced HMGA2 (Fig. 6C, *P* < 0.05).

To further clarify the role of HMGA2 in the formation of VM *in vivo*, we evaluated the expression of Twist1 and VE-cadherin by immunohistochemistry (Fig. 6D). Consistent with our results, increased Twist1 and VE-cadherin expression levels were observed in tumours with high HMGA2 expression, while the converse relationship was observed in tumours with shRNA-mediated silencing of HMGA2. Meanwhile, we evaluated the expression levels of E-cadherin and vimentin in the xenograft model via IHC staining. The results were consistent with our previous observations and demonstrated that upregulation of HMGA2 resulted in low E-cadherin expression and high vimentin expression in the xenografts, whereas downregulation of HMGA2 significantly enhanced the expression of E-cadherin and suppressed the expression of vimentin in xenografts of transfected MGC803 cells (Fig. 6D). Furthermore, HMGA2 promoted the expression of MMP2, which plays an important role in metastasis (Fig. 6D). Collectively, these findings indicate that HMGA2 promotes VM and metastasis by regulating the Twist1-VE-cadherin axis *in vivo*.

Taken together, our data suggest that HMGA2 directly targets Twist1 and promotes the expression of Twist1 and VE-cadherin, resulting in the formation of VM and enhanced tumour invasion and metastasis.

## Discussion

Angiogenesis is a hallmark of cancer and is critical for tumourigenesis. As such, anti-angiogenesis therapy has become an important approach as a viable treatment. While recent publications have shown that the development of endothelial-lined vessels is inhibited by anti-angiogenic agents, VM and metastasis increase despite this therapeutic approach. Thus, vascular mimicry is believed to play an important role in cancer progression and differs from the endothelial cells that normally develop into blood vessels^32, 33^. The occurrence of VM at an early stage of tumourigenesis is thought to represent a crucial step in tumour progression and metastasis^7–10^. Therefore, evaluating the therapeutic potential of inhibiting VM remains of critical clinical importance. HMGA2 is a transcription factor that plays an important role in a variety of cancers^34–37^, including GC. However, its contributions (if any) to VM remain unclear. In the present study, we provided evidence that HMGA2 expression correlates with VM density in GC and promotes VM in a xenograft model as well as in cell lines. In addition, we demonstrated that HMGA2 directly targets Twist1 at the DNA and protein level and increases the expression of Twist1 and VE-cadherin, thereby promoting the formation of VM. Thus, HMGA2 and VM are potential therapeutic targets for GC.

Across our cohort, nearly 40 of the gastric carcinoma cases demonstrated high HMGA2 protein expression. Tumours with elevated HMGA2 expression are associated with a poor prognosis and an elevated incidence of metastasis. HMGA2 was associated with tumour differentiation, TNM stage, metastasis and recurrence and positively correlated with VM.

In the present study, 28.5% of patients with gastric carcinoma displayed VM, an incidence comparable to previously reported results^9, 21^. VM was observed in almost 65% of gastric carcinomas with high HMGA2 expression. *In vivo and in vitro* experiments highlighted the role of HMGA2 in VM channel formation. Specifically, tumours with high levels of HMGA2 protein expression formed VM channels, whereas tumours with low HMGA2 expression did not present the same effect. Notably, VM correlated with tumour differentiation, TNM stage, metastasis and recurrence. We also found that the overall survival rate was much lower in patients with tumours positive for HMGA2 and VM. Our results suggest the involvement of HMGA2 in the formation of VM and promotion of disease progression.

VE-cadherin has been identified as a key marker for vasculogenic mimicry, which plays an important role in the formation of VM, because cells lacking VE-cadherin are incapable of forming VM tube^31, 38^. In the present study, we examined VE-cadherin expression with respect to the expression levels of HMGA2 in human GC cells. Our data show that when HMGA2 is expressed, VE-cadherin expression is upregulated at the DNA and protein level, while HMGA2 downregulation conversely leads to decreased VE-cadherin. Thus, the analysis of our patient cohort demonstrated that VE-cadherin expression positively correlates with HMGA2 in GC, and patients positive for HMGA2 and VE-cadherin have a poor prognosis. It is reasonable to propose that HMGA2 promotes VM formation in GC by regulating VE-cadherin. A computational analysis predicted that HMGA2 can target the promoter of Twist1^39^. It is known that Twist1 opens nuclear membrane pores with the help of an accessory protein and enters the nucleus to regulate the transcription of downstream genes that are involved in the process of VM^40^, and we have previously reported that Twist1 facilitates VM by modulating the promoter of VE-cadherin in hepatocellular carcinoma^30^.

To further demonstrate that HMGA2 promotes VM by targeting Twist1 to upregulate the expression of VE-cadherin, we first analysed the relationship between HMGA2 and Twist1, using a luciferase assay, ChIP and co-immunoprecipitation experiments. The results of these assays demonstrated that HMGA2 can directly target Twist1 in GC cell lines. Meanwhile, Twist1 expression is significantly upregulated in VM-positive GC tissues, and this upregulation is strongly correlated with HMGA2 overexpression. Subsequently, we silenced Twist1 in MKN74 cells that stably overexpresseed HMGA2. Our results indicate that VM formation and cell migration is reduced in these cells. When Twist1 expression is reduced, the HMGA2 protein levels were not changed, which is consistent with the above results. As our previous report demonstrated, we found that VE-cadherin is also reduced when Twist1 was downregulated in cells, resulting in inhibited VM and reduced cell migration, however, E-cadherin expression did not change. Moreover, Twist1 is associated with VM in GC, and our survival analysis demonstrated that GC patients with positive Twist1 and VE-cadherin expression have a worse prognosis compared with GC patients without Twist1 and VE-cadherin expression. These findings confirm that HMGA2 promotes VM by directly targeting Twist1 to regulate VE-cadherin.

EMT, which plays an important role in embryogenesis, wound healing, organ fibrosis, and cancer metastasis, is known to contribute to tumour cell plasticity and is also a characteristic of VM^10, 30^. As with VM, the epithelial-to-mesenchymal transition heavily depends on the capacity of tumour cells to gain a trans- or de-differentiated phenotype and a set of transcription factors, such as Twist and Snail as well as to coordinate EMT and related migratory processes. To comprehensively characterize the effects of HMGA2 on VM, we evaluated the expression of EMT markers in cells transfected with either HMGA2 plasmid or sh-HMGA2 plasmid. Our results demonstrate that in MKN74 and MKN28 cells, increased HMGA2 expression significantly enhances the expression of the mesenchymal markers vimentin and N-cadherin and decreased the expression of the epithelial marker E-cadherin. Downregulation of HMGA2 significantly suppressed vimentin and N-cadherin but induced E-cadherin expression in MGC803 and MKN45 cells, which may explain why E-cadherin expression did not increase when we silenced Twist1 in MKN74 cells that overexpressed HMGA2. This result is consistent with reports that HMGA2 epigenetically silences the E-cadherin gene during the epithelial-to-mesenchymal transition^41^. Animal models also show that HMGA2 is associated with EMT, and clinical data indicate that HMGA2, E-cadherin and vimentin influence the prognosis of patients with GC. The presence of these features within a tumour is related to increased tumour malignancy, and this association is well documented for VM.

VM plays an important role in tumour growth and metastasis^8–10, 42, 43^. In the present study, we provide evidence that VM is associated with HMGA2 expression. Moreover, we found that upregulation of HMGA2 promoted the invasion and metastasis of GC cells and correlated with metastasis in our clinical cohort. Second, HMGA2 is related to MMP2 in GC. Third, MMP expression and activity were upregulated after HMGA2 upregulation. Indeed, HMGA2 knockdown resulted in reduced tumour cell migration and network formation *in vitro* and reduced the activity of MMP. Furthermore, in the xenograft mouse models, tumours derived from HMGA2 overexpressing cells were much larger than those form the control group. This growth-promoting role of HMGA2 may result from increased proliferation. Thus, it is plausible to consider that HMGA2 promotes metastasis of gastric carcinoma via induction of VM.

In conclusion, we demonstrate that HMGA2 is correlated with GC VM formation and that positivity for both HMGA2 and VM predicts a worse clinical outcome for GC patients. HMGA2 can directly target Twist1 and promote the expression of Twist1 and VE-cadherin, thereby inducing VM—this indicates that HMGA2 and this pathway are potential novel therapeutic targets for GC.

## Electronic supplementary material

supplementary info
