# Supplementary material for: HMGA2 promotes vasculogenic mimicry and tumor aggressiveness by upregulating Twist1 in gastric carcinoma
Source: Sci Rep. 2017 May 22;7:2229. doi: 10.1038/s41598-017-02494-6 (PMC5440402; doi:10.1038/s41598-017-02494-6)
Supplement: Supplementary file 1 — supplementary info [file 41598_2017_2494_MOESM1_ESM.pdf]

HMGA2 promotes vasculogenic mimicry and tumor aggressiveness by upregulating Twist1 in gastric carcinoma

Junying Sun[1,#],Baocun Sun[1,2,3,#]*,Ran Sun[4],Dongwang Zhu[5],Xiulan Zhao[1,2], Yanhui Zhang[3], Xueyi Dong[1],Na Che[1,2],Jing Li[1,2],Fang Liu[1],Nan Zhao[1,2], Yong Wang[3], Danfang Zhang[1,2]

[1] Department of Pathology, Tianjin Medical University, Tianjin 300070, China

[2] Department of Pathology, Tianjin General Hospital, Tianjin Medical University, Tianjin 300052, China

[3] Department of Pathology, Tianjin Cancer Hospital, Tianjin Medical University, Tianjin 300060, China

[4] Department of Surgery, Tianjin Nankai Hospital, Tianjin 300100, China

[5] Department of Prosthodontics, Affiliated Stomatological Hospital, Tianjin Medical University, Tianjin 300070, China

#These authors equally contribute to this study.

***Corresponding author:**

Prof. Baocun Sun，

Department of Pathology and General Hospital and Cancer Hospital of Tianjin Medical University, Tianjin 300070, PR China;

E-mail: baocunsun@aliyun.com

Tel: 86-13602111192

Fax: 86-22-83336813

Mailing Address： No.22 Qixiangtai Road, Heping District, Tianjin 300070, China

Table S1. Antibodies used in this study

**Antibody**	**Source**	**IHC Concentration/ WB**	**Product Number**	**Manufacture**
HMGA2	Rabbit	1:100 (IHC)<br>1:500(WB)	Ab-52039	Abcam
Twist1	Rabbit	1:200 (IHC)<br>1:200(WB)	Sc-15393	Santa Cruz
VE-cadherin	Rabbit	1:500(WB)<br>1:400(IHC)	ab33168	Abcam
E-cadherin	Mouse	1:200(WB)<br>1:100(IHC)	ab1416	Abcam
N-cadherin	Rabbit	1:1000(WB)	AF4039	Affinity Biosciences
Vimentin	Rabbit	1:500(WB)<br>1:200(IHC)	ab92547	Abcam
MMP2	Rabbit	1:200(WB)<br>1:100(IHC)	10373-2-AP	LuoSai-BIO
MMP9	Rabbit	1:500(WB)	ab76003	Abcam
GAPDH	Rabbit	1:2000(WB)	Sc-25778	Santa Cruz
CD34	Mouse	1:550(IHC)	Zm-0046	ZSGB-Bio
Endomucin	Rat	1:800(IHC)	14-5851-81	Ebioscience

Note: IHC: Immunohistochemistry, WB: Western blot.

Table S2. The sequences of primer used for Semi-quantitative RT-PCR

Gene	Forward primer(5’-3’)	Reverse primer(5’-3’)
Twist1	CCATCCACACCGTCCCCTCCCCCTC	TCTGGCTCTTCCTCGCTGTTGCT
CDH5	ACAAGGACATAACACCACGAAACG	TGAGATGACCACGGGTAGGAAGT
HMGA2	CCCAAAGGCAGCAAAAACA A	GCCTCTTGGCCGTTTTTCTC
GAPDH	CCTGGCCAAGGTCATCCATGAC	TGTCATACCAGGAAATGAGCTTG

Table S3. The sequences of primer used for ChIP-q PCR

**Primer name**	**Forward primer (5′-3′)**	**Reverse primer (5′-3′)**
Primer1	CCCACTGTGTAGAAGCTGTTG	GAAGCGGTCGGAGGAGA
Primer2	GTCACAATGCGGAGCCTAAT	AAACCCAGTCCATGGGAAAG
Primer3	GGTTAATGGGCAGCTAAGACA	CCTGTAGAAAGTGCAGGATCAG
Primer4	CTCATGATGTCAGGCGGTTT	ACGGAGTTCTGTTTGGGTTTC
Primer5	GCGGTGCCAAGTGTCAG	GTCTCCGGAAATTCGAGGTTG
Primer6	GAAGGTGTGTAATTGGAGTTTAAGG	CTCCTCTGTGCCAGTGATTT

Fig.S1 The expression of HMGA2 in GC cell lines and its relation of Twist1, VE-cadherin in GC patients.

(A)Western blot showed the expression of HMGA2 expression in various GC cell lines (*p < 0.05) (B) Immunofluorescence staining showed the expression of HMGA2 in cells which formed VM on matrigel.(C)The relation of HGMA2 and Twist1, VE-cadherin in GC patients and they are significantly related to poor prognosis (*P<0.05)

Fig.S1

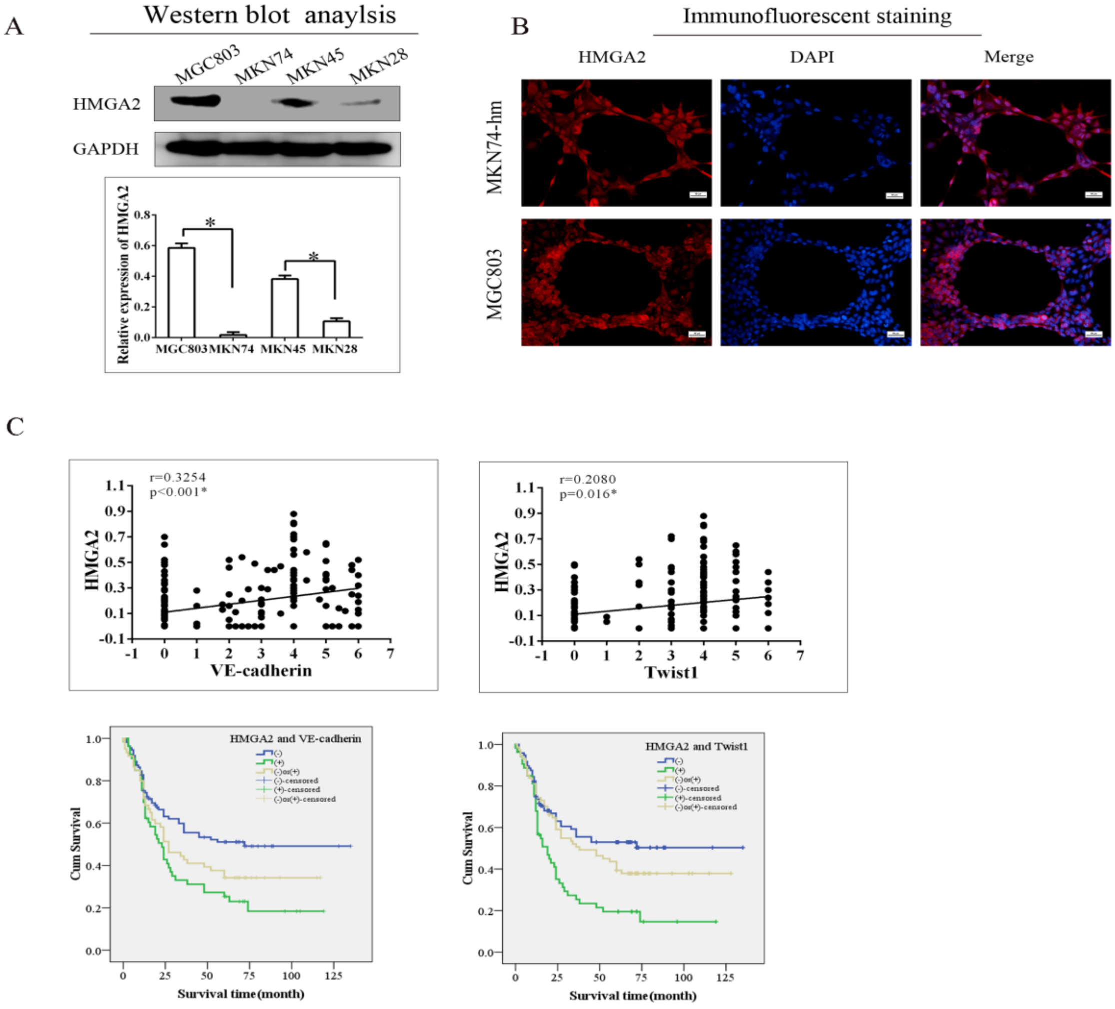

A
Western blot anaylsis
MGC803
MKN74
MKN45
MKN28
HMGA2
GAPDH
Relative expression of HMGA2
0.8
0.6
0.4
0.2
0.0
*
*
MGC803
MKN74
MKN45
MKN28
B
Immunofluorescent staining
HMGA2
DAPI
Merge
MKN74-hm
MGC803
C
r=0.3254
p<0.001*
HMGA2
VE-cadherin
r=0.2080
p=0.016*
HMGA2
Twist1
HMGA2 and VE-cadherin
Cum Survival
Survival time(month)
HMGA2 and Twist1
Cum Survival
Survival time(month)

Fig.S2

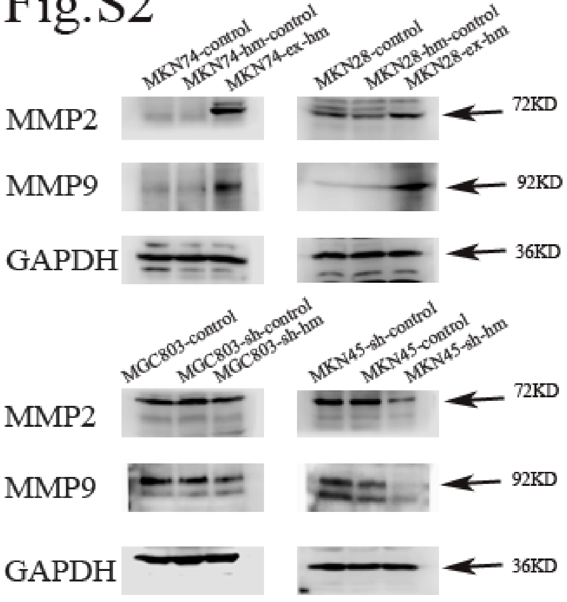
MKN74-control
MKN74-hm-control
MKN74-ex-hm
MKN28-control
MKN28-hm-control
MKN28-ex-hm
MMP2
72KD
MMP9
92KD
GAPDH
36KD
MGC803-control
MGC803-sh-control
MGC803-sh-hm
MKN45-sh-control
MKN45-control
MKN45-sh-hm
MMP2
72KD
MMP9
92KD
GAPDH
36KD

Fig.S3

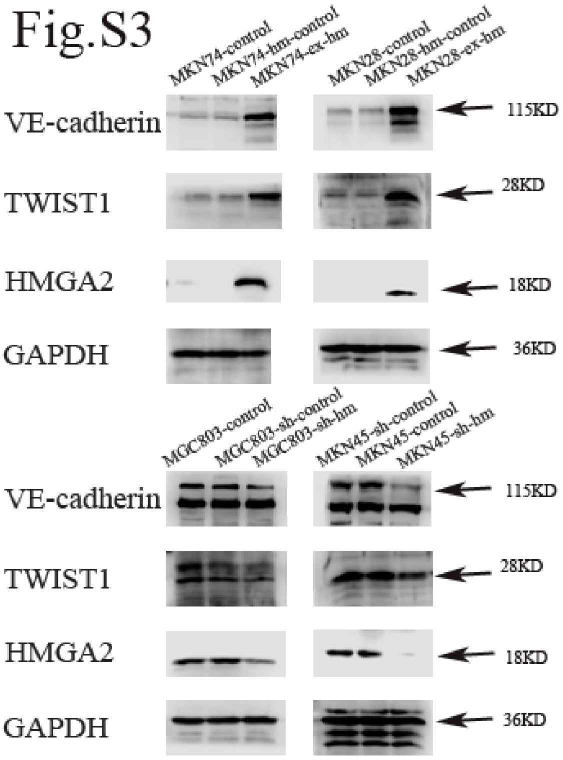
MKN74-control
MKN74-hm-control
MKN74-ex-hm
MKN28-control
MKN28-hm-control
MKN28-ex-hm
VE-cadherin
115KD
TWIST1
28KD
HMGA2
18KD
GAPDH
36KD
MGC803-control
MGC803-sh-control
MGC803-sh-hm
MKN45-sh-control
MKN45-control
MKN45-sh-hm
VE-cadherin
115KD
TWIST1
28KD
HMGA2
18KD
GAPDH
36KD

Fig S4

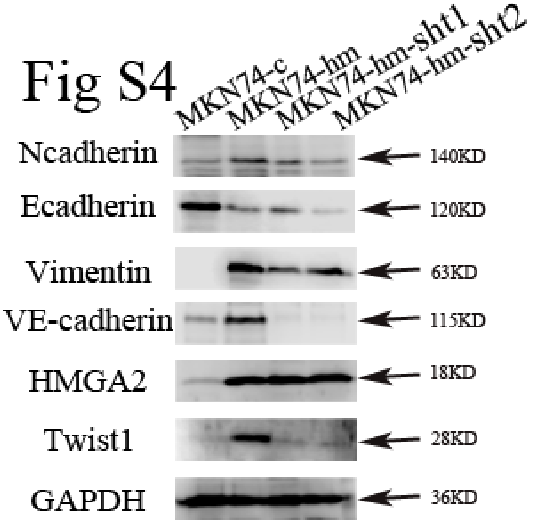
MKN74-c
MKN74-hm
MKN74-hm-sht1
MKN74-hm-sht2
Ncadherin
140KD
Ecadherin
120KD
Vimentin
63KD
VE-cadherin
115KD
HMGA2
18KD
Twist1
28KD
GAPDH
36KD

Fig S5

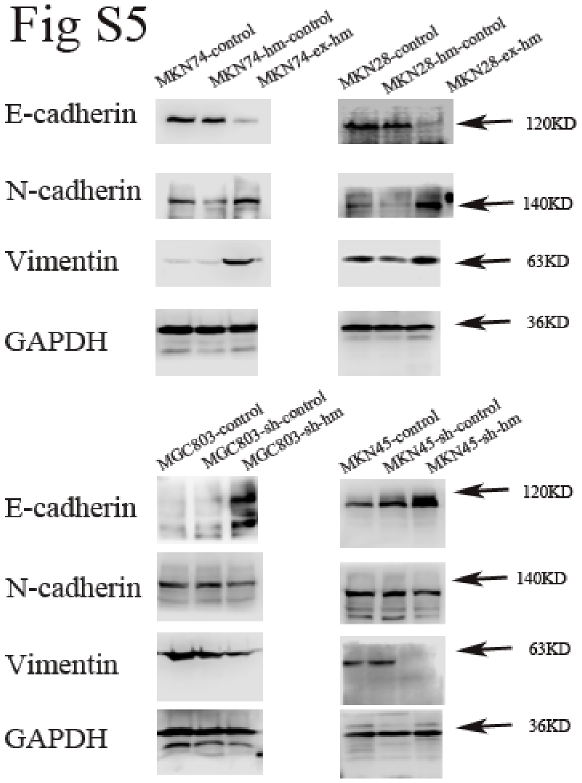
MKN74-control
MKN74-hm-control
MKN74-ex-hm
MKN28-control
MKN28-hm-control
MKN28-ex-hm
E-cadherin
120KD
N-cadherin
140KD
Vimentin
63KD
GAPDH
36KD
MGC803-control
MGC803-sh-control
MGC803-sh-hm
MKN45-control
MKN45-sh-control
MKN45-sh-hm
E-cadherin
120KD
N-cadherin
140KD
Vimentin
63KD
GAPDH
36KD
